# A case of EBV encephalomyelitis with positive anti-GFAP-IgG antibody with recurrent fever and dysuresia as the main symptoms: Case report and retrospective analysis

**DOI:** 10.1097/MD.0000000000031995

**Published:** 2022-12-02

**Authors:** Lulu Wang, Lulu Dong, Mingmin Zhao, Chao Jiang, Minxia Geng, Shuang Li, Jiahao Xing, Tianjun Wang

**Affiliations:** a Department of Neurology, Hebei General Hospital, Shijiazhuang, Hebei, China; b Graduate School of North China University of Science and Technology, Tangshan, Hebei, China; c Graduate School of Hebei North University, Zhangjiakou, Hebei, China; d Graduate School of Hebei Medical University, Shijiazhuang, Hebei, China.

**Keywords:** CSF, encephalomyelitis, Epstein-Barr virus, genetic testing, glial fibrillary acidic protein

## Abstract

Due to neuronal destruction caused by Epstein-Barr virus (EBV) infection, exposure to neuronal surface antigens may lead to an imbalance in immune tolerance, which in turn triggers an autoimmune response. In addition, due to the involvement of nonspecific B-cell activation or molecular mimicry, EBV and Glial Fibrillary Acidic Protein (GFAP) receptors may have common epitopes. Viral infection triggers activation of B-cell and cross-reaction with viral antibodies, resulting in autoimmune encephalomyelitis. The clinical presentation may be complex or even atypical. A small number of patients may develop autoimmune reactions.

**Patient concerns:**

Most patients with EBV encephalomyelitis have a good prognosis, with the disease generally having a short course, few complications, and a good prognosis. In most patients, after treatment, their neurological function basically recovers within a few weeks or months.

**Diagnosis interventions:**

The patient had fever and headache. His 3 tests for cerebral spinal fluid (CSF) are consistent with the features of viral encephalomyelitis. Pathogenic examination of CSF confirmed EBV, and imaging suggested brain and spinal cord involvement. After antiviral treatment, the patient’s symptoms relieved. The diagnosis of EBV encephalomyelitis was considered. However, the patient’s temperature continued to increase. He was transferred to a superior hospital and was given GFAP-Ab in CSF, which was strongly positive. The patient was given immunoglobulin and antiviral therapy. This supports the diagnosis of GFAP-IgG antibody positive with EBV encephalomyelitis.

**Outcomes:**

After treatment with antiviral drugs and immunoglobulins, the patient’s symptoms improved and he was able to function.

**Lessons:**

EBV encephalomyelitis is a rare clinical disease. Therefore, more attention should be paid to the early diagnosis and treatment of similar patients to avoid misdiagnosis. CSF tests, genetic tests, and imaging tests can confirm the diagnosis.

## 1. Introduction

The antibody of glial fibrillary acidic protein (GFAP) is produced by infiltrating lymphocytes in the peripheral and central nervous system, which is considered as a biomarker of autoimmune GFAP astrocytic disease. The brain, meninges, spinal cord and optic nerve are the most susceptible and sensitive to hormone.^[1]^ Encephalomyelitis caused by Epstein-Barr virus (EBV) infection is a serious infectious disease of the central nervous system. Autoimmune reactions secondary to EBV encephalomyelitis are rare and should be of concern to neurologists. This article reports a relatively rare case of a patient with EBV encephalomyelitis complicated with GFAP-IgG antibody positive. Thus, making a retrospective analysis of diagnosis and treatment with the literature to further understand this disease.

## 2. Case presentation

**Figure 1.**
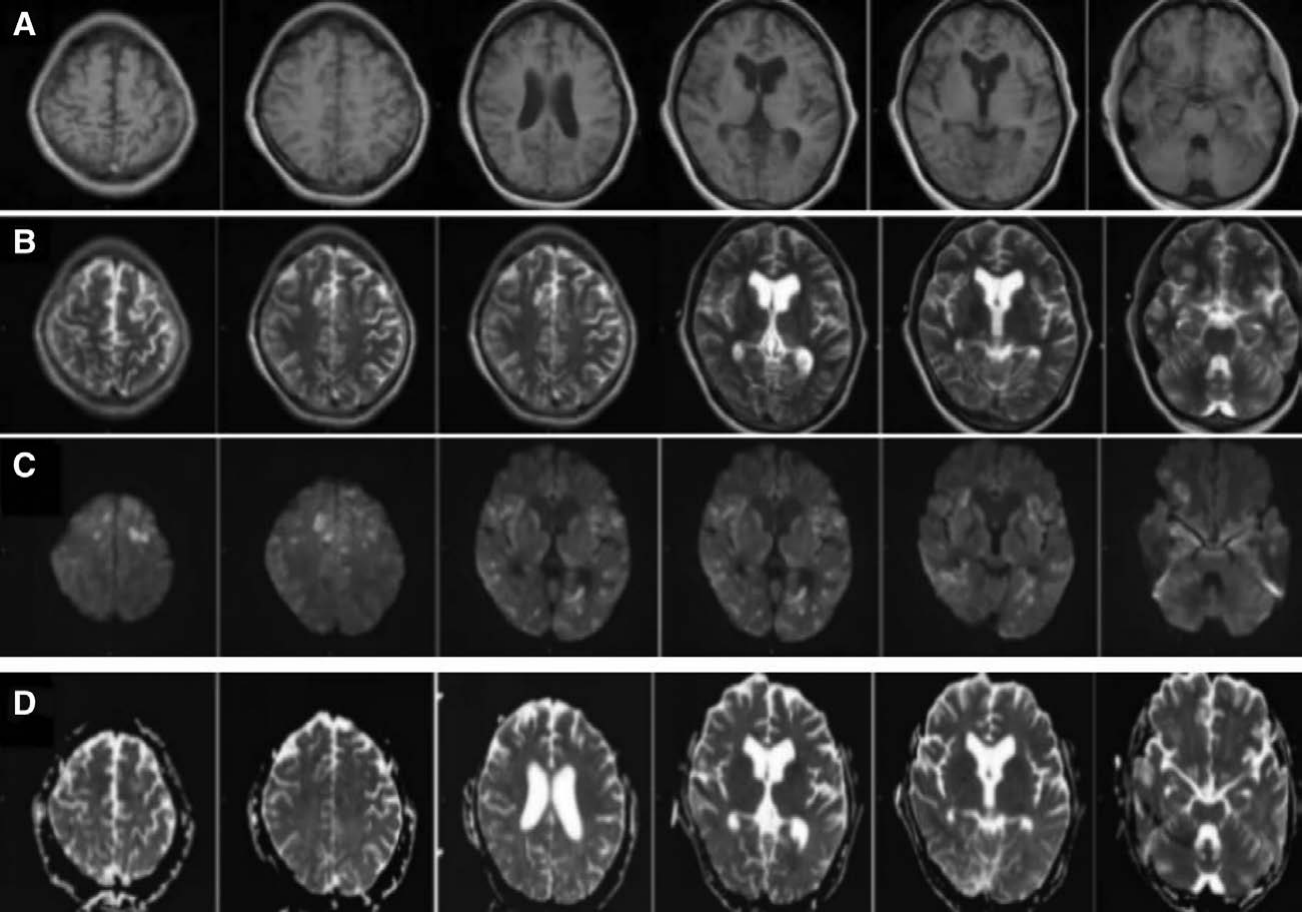
The cortex and white matter in both cerebral hemispheres had long T1 and T2 signals and a high signal in the FLAIR image. The lesion in DWI with high signal intensity and ADC with low signal intensity.

**Figure 2.**
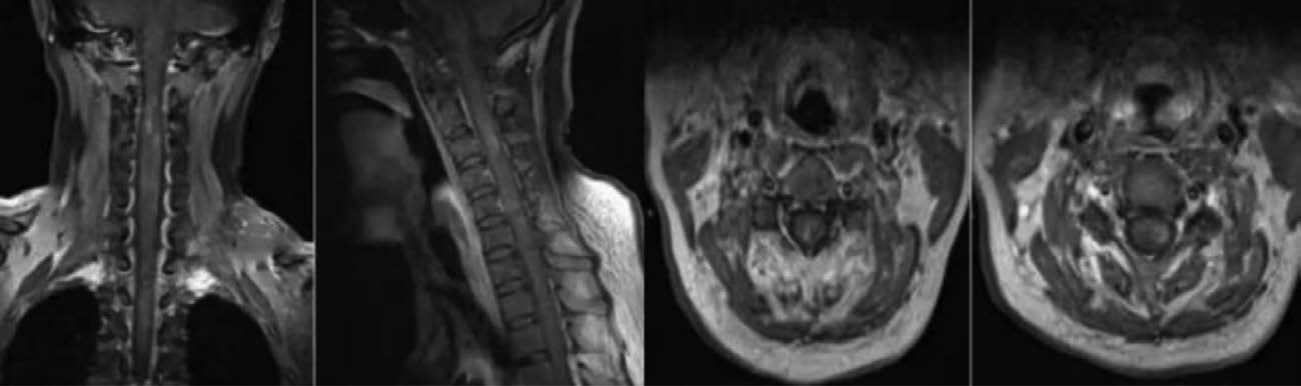
MRI of the cervical spine showed significant signal changes with enhanced and patchy C2-5 levels.

**Figure 3.**
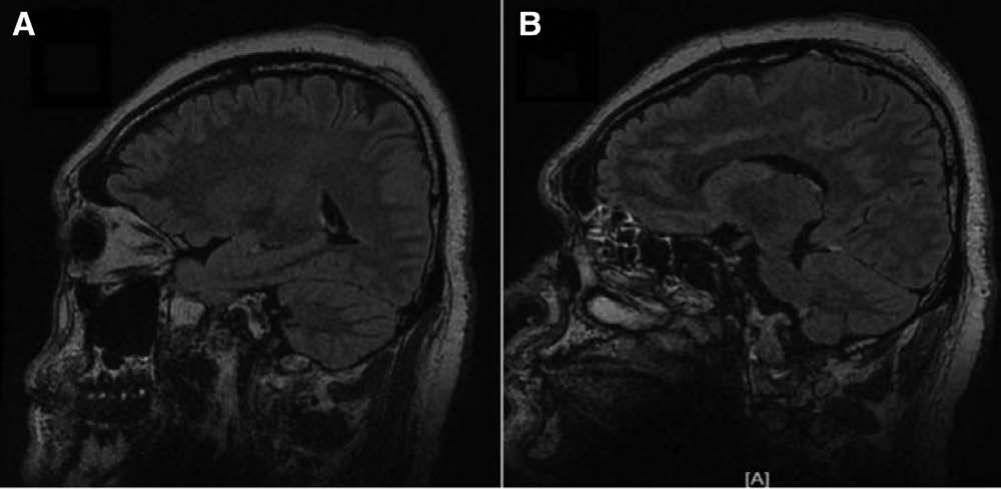
The CUBE enhancement of meninges indicates significant enhancement of the left tentorium cerebellum and left posterior central gyrus cortex.

**Figure 4.**
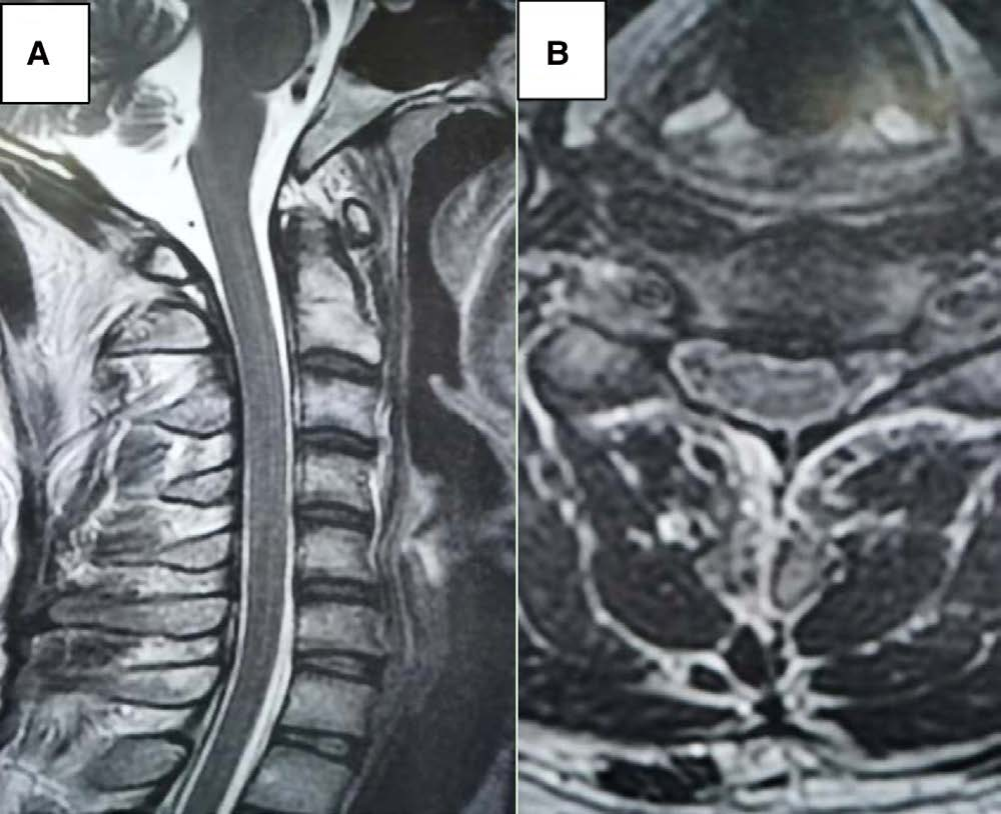
In PET images, diffusivity increases throughout the spinal cord in heterozygous metabolism.

One week prior to hospitalization, a 37-year-old male developed a fever and headache. The patient’s body temperature was up to 39°C, accompanied by shivering, and then he developed throbbing headache. The location of the pain was not known, and the pain score was 3. During the course of the disease, the patient has recurrent fever, no cough and sore throat, poor spirits, poor diet and sleep, and normal urine and feces. She was in good health, had caught a cold 2 days before the attack after a business trip, and had no particular family history. Physical examination: The main positive signs of the patient were as follows: positive limb tendon reflex, positive neck resistance, talar 3 transverse fingers. Examination after admission includes 7 blood analysis (Table 1), 3 lumbar puncture (Table 2). The urine and stool analysis; thyroid function; all male tumor items; 5 blood clotting items; 8 preoperative items; ten tips for serum virus; respiratory pathogen profile; biochemical items; rheumatoid items; erythrocyte sedimentation rates; cerebral spinal fluid (CSF) ink stain; Gram stain; acid-fast stain; echocardiogram:normal. The EBV DNA of CSF detected by real-time PCR was 2.68 × 103 copies/mL (normal reference range < 500 copies/mL). Human herpesvirus type 4 (EBV) was detected by metagenomic second-generation sequencing technology in CSF samples: 22 specific sequences with high confidence intervals. Other antibodies, such as anti-aquaporin 4, myelin oligodendrocyte glycoprotein antibody, GFAP antibody were negative. The brain MRI (Fig. 1) showed that the cortex and white matter in both cerebral hemispheres had long T1 and T2 signals and a high signal in the FLAIR image. The lesion in DWI with high signal intensity and ADC with low signal intensity. In addition, MRI of the cervical spine (Fig. 2) showed significant signal changes with enhanced and patchy C2-5 levels. The CUBE enhancement of meninges (Fig. 3) indicates significant enhancement of the left tentorium cerebellum and left posterior central gyrus cortex. In PET images (Fig. 4), diffusivity increases throughout the spinal cord in heterozygous metabolism. Diagnosis and treatment process: In patients with acute symptoms such as fever and headache. His 3 tests for CSF were consistent with the features of viral encephalomyelitis. Pathogenic examination of CSF confirmed EBV, and imaging suggested brain and spinal cord involvement. On the fourth day after admission, the patient developed complications such as dysuresia. After antiviral treatment, the patient’s symptoms relieved. Thus, the overall clinical picture suggested the diagnosis of EBV encephalomyelitis. However, the patient’s temperature continued to increase after antiviral treatment with 0.375g of ganciclovir every 12 hours. Since autoimmune diseases had not been ruled out, immunoglobulin (0.4 g/kg-1d-1) was temporarily applied, and then the patient’s body temperature gradually decreased to normal. On the 15th day after admission, the patient’s temperature rose to 38.1°C again, and then he was transferred to a higher hospital for diagnosis and treatment. CSF GFAP-AB was strongly positive and the patient was again given immunoglobulin and antiviral therapy. The patient gradually improved.

**Table 1:** Blood tests before and after admission results.

Time	White blood cell count (×10^9^/L)	Neutrophils (%)	Lymphocytes (%)	Monocytes (%)	Eosinophilic (%)	Hemoglobin (g/L)
Before 1 wk	11.44	64.3	-	-	-	-
After 1 d	12.79	72.40	14.90	-	-	169.00
After 3 d	8.53	64.10	23.80	4.2	1.80	147.00
After 5 d	10.25	69.90	21.60	6.3	0.70	135.00
After 8 d	9.62	75.60	19.10	4.5	0.10	137.00
After 11 d	12.12	72.60	23.50	2.6	0.10	140.00
After 15 d	13.05	67.80	26.70	4.1	0.20	147.00

**Table 2:** CSF before and after admission results.

Time	Pressure (mmH2O)	Cell population (×10^9^/L)	Total protein (mg/L)	Glucose (mmol/L)	Chloride (mmol/L)
After 7 d	270	74.00	56.44	44.8	116
After 10 d	180	137.00	78.31	42.44	117
After 12 d	180	48.00	35.38	41.97	118
After transfer	160	46.00	46.69	43.27	117

CSF = cerebral spinal fluid.

## 3. Discussion

EBV, also known as Human Herpesvirus 4, can directly invade the nervous system, such as the meninges, brain, spinal cord and peripheral nerves.^[2]^ EBV causes central system infection through 2 mechanisms. First, EBV infects B lymphocytes through the interaction of the viral glycoprotein GP350/220 with the neural cell receptor CD21.^[3]^ EBV DNA can exist outside the host chromosome or be incorporated into the host cell genome.^[4]^ Second, EBV not only releases toxins directly into the nervous system through the immune-mediated effect of CD8 + T lymphocytes, but also produces antigen-antibody complexes that can cause brain cell damage. EBV is the causative agent of infectious mononucleosis and has also been linked to a variety of diseases, such as childhood lymphoma and nasopharyngeal carcinoma. EBV infection can cause encephalitis, meningitis and myelitis, of which encephalitis and meningitis are more common and myelitis is rare.^[5]^ Most patients with EBV encephalomyelitis have an acute onset and a variety of clinical manifestations, including headache, fever, limb weakness, muscle twitching, etc.^[6]^ Other studies have reported neurological complications following EBV infection, including Guillain-Barre syndrome, demyelinating cerebrospinal disease, transverse encephalomyelitis, and multiple myelopathy.^[7]^ PCR tests for EBV DNA in CSF are sensitive and specific, and have been used as the first choice for the diagnosis of EBV central nervous system infection.^[8]^ In addition, CSF examination can be used as one of the criteria to distinguish EBV encephalomyelitis from other encephalomyelitis. CSF in EBV encephalomyelitis is characterized by increased pressure and white blood cell counts, mainly lymphocytosis, a slight increased in protein, and normal sugar and chloride content.^[9]^ The disease generally has a short course, few complications, and a good prognosis.

In this case, next-generation sequencing of CSF showed a high fiducial interval for EBV, but GFAP-IgG antibodies in the CSF were negative. However, GFAP-IgG antibodies were strongly positive when transferred to higher hospital. We furthermore wondered whether initially negative GFAP-IgG antibodies were a preclinical manifestation of EBV encephalomyelitis. As the course of the disease prolongs, the GFAP-specific IgG antibodies have the potential to turn positive. It has been reported that the development of EBV encephalomyelitis can be associated with autoimmune reactions, such as GFAP astrocytic disease. Rutkowska et al^[10]^ found that EBV inducible gene 2 (EBI2) activation in astrocytes could stimulate extracellular signal-regulated kinase phosphorylation and Ca^2 + ^signal transduction pathway to induce cell migration through EBV-induced gene 2 knockout mice. This is the first time that EBI2 has been shown to regulate relevant receptors in astrocytes, which play an essential role in the diagnosis and treatment of central nervous system infectious diseases. GFAP is primarily found in astrocytes in the central nervous system.^[11]^ It plays an essential role in the formation of the cytoskeleton, the maintenance of cell morphology, the regulation of cell synaptic function, and the maintenance of the integrity of the blood-brain barrier. Therefore, we hypothesize that GFAP sets the stage for EBI2 activation and receptor regulation by participating in astrocyte formation.^[12]^ We, therefore, analyzed the possibility that the positive GFAP-IgG antibody in this patient was triggered by EBV infection.

GFAP astrocytosis is an autoimmune disease of the central nervous system that can be caused by viral or tumor infections.^[13]^ The etiology is unclear. Positive serum or CSF GFAP-specific IgG antibodies are a specific indicator for the diagnosis of autoimmune GFAP astrocytosis. It is frequently accompanied by additional autoantibodies, such as N-methyl-D-aspartate receptor antibodies, Aquaporin4 antibodies, etc.^[14]^ The age of onset of GFAP astrocytosis tends to be over 40 years, and the majority of patients are women. Clinical features of the disease include fever, headache, loss of vision, chronic encephalomyelitis and autonomic dysfunction. MRI reveals multiple lesions, often involving white matter, basal ganglia, brainstem, cerebellum, meninges, etc. These are characteristic changes of autoimmune GFAP encephalomyelitis. The condition can be distinguished from Neuromyelitis optica spectrum disorder. People with NMOSD often have optic nerve damage and transverse myelitis, and have recurrent episodes of the disease.^[15]^ MRI of the spinal cord indicates that the lesion is more than 3 spinal cord segments, and the NMO-IgG antibody is positive.^[16]^ The features of the disease did not agree with this patient, and were therefore excluded. In this case, MRI of the brain revealed multiple lesions in both hemispheres of the brain, and PET of the cephalic spinal cord revealed increased inhomogeneous metabolism throughout the spinal cord.

Several studies have shown that viral infection is the mechanism of autoimmune encephalomyelitis. Currently, related viruses include herpes simplex virus, influenza virus, EBV, etc, of which herpes simplex virus is the most common.^[17]^ This case was secondary to EBV infection with GFAP-IgG antibody-positive encephalomyelitis, which is relatively rare in clinical practice. The pathogenesis of EBV infection may be due to neuronal destruction caused by EBV infection, where exposure to neuronal surface antigens may lead to an imbalance in immune tolerance, which then triggers an autoimmune response. In addition, due to the involvement of nonspecific B-cell activation or molecular mimicry, EBV and GFAP receptors may have common epitopes. Viral infection triggers activation of B cells and cross-reaction with viral antibodies, resulting in autoimmune encephalomyelitis.^[18]^ Related literature reports that about 70% of patients respond well to hormone therapy, and some patients are prone to relapses.

Currently, there is no specific treatment for EBV encephalomyelitis, and related literature reports can be reconciled with the treatment of EBV encephalitis. The antiviral drug ganciclovir can inhibit EBV replication, but there is no evidence to support the use of antiviral drugs for EBV-associated diseases. Currently, the use of adrenocortical hormones remains controversial. Adachi et al^[19]^ found that the hormone is effective for EBV patients with encephalomyelitis, but it needs long-term application, and the drug withdrawal will cause relapse. According to the literature, once infected with EBV, it remains latent in human B lymphocytes for life, and EBV has acquired the ability to disrupt or evade immune surveillance. Eveline et al^[20]^ found that early genes of BamHI-A Rightward openreading Frame 1 region encoded by EBV could create microenvironment in immune evasion. Tumor cells were killed by encoding granulocyte-macrophage colony-stimulating factor. Ke et al^[21]^ found that intravenous or combination intrathecal administration of rituximab achieved good efficacy, and the reason may be related to the opening of the blood-brain barrier caused by inflammation, which favors rituximab penetration. In addition, nutritional nerve cell drugs and functional rehabilitation therapy are also essential.

In summary, EBV infection should be considered in clinical practice when encountering brain and long-segment spinal cord lesions due to intracranial infection. Currently, the association between autoimmune GFAP astrocytic disease and EBV encephalomyelitis and treatment regimens has not been clarified, suggesting that we should continue to search for and summarize in future clinical work.

## Acknowledgments

The authors would like to thank Tianjun-Wang for her assistance in writing this manuscript.

## Author contributions

All the authors contributed equally to this work. All authors have read and approved the final manuscript.

**Resources:** Lulu Dong, Mingmin Zhao, Chao Jiang, Minxia Geng, Shuang Li, Jiahao Xing.

**Writing – original draft:** Lulu Wang.

**Writing – review & editing:** Tianjun Wang.

## References

[1] Yang Z Wang K. Glial fibrillary acidic protein: from intermediate filament assembly and gliosis to neurobiomarker. Trends Neurosci. 2015;38:364–74. 25975510 10.1016/j.tins.2015.04.003 PMC4559283

[2] Jonathan R. Epstein-Barr virus (EBV) reactivation and therapeutic inhibitors. J Clin Pathol. 2019;72:651–8. 31315893 10.1136/jclinpath-2019-205822

[3] Zhang N Zuo Y Jiang L. Epstein-Barr virus and neurological diseases. Front Mol Biosci. 2022;10:816098. 10.3389/fmolb.2021.816098 PMC8784775 35083281

[4] Dyachenko P Smiianova O Kurhanskaya V. Epstein-Barr virus-associated encephalitis in a case-series of more than 40 patients. Wiad Lek. 2018;71:1224–30. 30267504

[5] Bruna K Douglas K. Viral encephalitis: a practical review on diagnostic approach and treatment. J Pediatr (Rio J). 2020;96:12–9. 31513761 10.1016/j.jped.2019.07.006 PMC9431993

[6] Andrew N Michael G. Epstein-Barr virus. Microbiol Spectr. 2016;4. 10.1128/microbiolspec.DMIH2-0011-2015 27337443

[7] Derler F Seidel S Bengel D. Fulminante EBV-meningoenzephalitis: gutes klinisches outcome bei einer jungen, immunkompetenten frau. Der Nervenarzt. 2017;88:1186–91. 28730393 10.1007/s00115-017-0381-4

[8] Wang Y Yang J Wen Y. Lessons from Epstein-Barr virus DNA detection in cerebral spinal fluid as a diagnostic tool for EBV-induced central nervous system dysfunction among HIV-positive patients. Biomed Pharmacother. 2022;145:112392. 34781140 10.1016/j.biopha.2021.112392

[9] Lupia T Milia LM Atzori C. Presence of Epstein-Barr virus DNA in cerebral spinal fluid is associated with greater HIV RNA and inflammation. AIDS. 2020;34:373–80. 31764071 10.1097/QAD.0000000000002442 PMC7773520

[10] Rutkowska A Preuss I Gessier F. EBI2 regulates intracellular signaling and migration in human astrocyte. Glia. 2015;63:341–51. 25297897 10.1002/glia.22757

[11] Elly M Milos P. Glial fibrillary acidic protein (GFAP) and the astrocyte intermediate filament system in diseases of the central nervous system. Curr Opin Cell Biol. 2015;32:121–30. 25726916 10.1016/j.ceb.2015.02.004

[12] Barington L Wanke F Arfelt K. EBI2 in splenic and local immune responses and in autoimmunity. J Leukoc Biol. 2018;104:313–22. 29741800 10.1002/JLB.2VMR1217-510R

[13] Li D Liu X Liu T. Neurochemical regulation of the expression and function of glial fibrillary acidic protein in astrocytes. Glia. 2020;68:878–97. 31626364 10.1002/glia.23734

[14] Kunchok A Zekeridou A McKeon A. Autoimmune glial fibrillary acidic protein astrocytopathy. Curr Opin Neurol. 2019;32:452–8. 30724768 10.1097/WCO.0000000000000676 PMC6522205

[15] Brian G Dean M. Neuromyelitis spectrum disorders. Mayo Clin. 2017;92:663–79. 10.1016/j.mayocp.2016.12.014 28385199

[16] Susanna A Graham C Friedemann P. Pain in NMOSD and MOGAD: a systematic literature review of pathophysiology, symptoms, and current treatment strategies. Front Neurol. 2020;21:778. 10.3389/fneur.2020.00778 PMC7812141 33473247

[17] Angelika O Cornel F Xandra O. Herpes simplex virus type 1 amplicons and their hybrid virus partners, EBV, AAV, and retrovirus. Curr Gene Ther. 2004;4:385–408. 15578989 10.2174/1566523043346129

[18] Bert A. A tolerogenic role of cathepsin G in a primate model of multiple sclerosis: abrogation by Epstein-Barr virus infection. Arch Immunol Ther Exp. 2020;68:21. 10.1007/s00005-020-00587-1 PMC7299916 32556812

[19] Adachi K Iwasaki S Tuji T. A case of meningoencephalitis caused by persistent Epstein-Barr (E-B) virus infection. Rinsho Shinkeigaku. 1989;29:89–92. 2545401

[20] Hoebe E Large T Tarbouriech N. Epstein-Barr virus-encoded BARF1 protein is a decoy receptor for macrophage colony stimulating factor and interferes with macrophage differentiation and activation. Viral Immunol. 2012;25:461–70. 23061794 10.1089/vim.2012.0034

[21] Ke P Ma X Bao X. Clinical analysis of 7 patients with Epstein-Barr virus encephalitis after allogeneic hematopoietic stem cell transplantation. Zhonghua Xue Ye Xue Za Zhi. 2017;38:685–9. 28954347 10.3760/cma.j.issn.0253-2727.2017.08.007 PMC7348247

